# Microbial Succession during Thermophilic Digestion: The Potential of *Methanosarcina* sp

**DOI:** 10.1371/journal.pone.0086967

**Published:** 2014-02-19

**Authors:** Paul Illmer, Christoph Reitschuler, Andreas Otto Wagner, Thomas Schwarzenauer, Philipp Lins

**Affiliations:** University Innsbruck, Institute of Microbiology, Innsbruck, Austria; Missouri University of Science and Technology, United States of America

## Abstract

A distinct succession from a hydrolytic to a hydrogeno- and acetotrophic community was well documented by DGGE (denaturing gradient gel electrophoresis) and dHPLC (denaturing high performance liquid chromatography), and confirmed by qPCR (quantitative PCR) measurements and DNA sequence analyses. We could prove that *Methanosarcina thermophila* has been the most important key player during the investigated anaerobic digestion process. This organism was able to terminate a stagnation phase, most probable caused by a decreased pH and accumulated acetic acid following an initial hydrolytic stage. The lack in *Methanosarcina* sp. could not be compensated by high numbers of *Methanothermobacter* sp. or *Methanoculleus* sp., which were predominant during the initial or during the stagnation phase of the fermentation, respectively.

## Introduction

Irrespective of the disputed contribution of man to the global warming, the dramatic effects *per se* and the involvement of gases like CO_2_ and CH_4_ are unquestionable. Therefore it has (or should have) become an important global goal to reduce uncontrolled greenhouse gas emissions. One possibility to do so (and to fulfill the Kyoto Protocol) is to increase the portion of renewable energy sources like biogas. Therefore and before the background of a decreasing availability and increasing costs of fossil energy sources, the European Union has decided that by the year 2020 about 5% of the total energy budget should be derived from biogas production (De Vrieze et al, 2012; EC, 2011). No wonder that both, the number and capacity of biogas plants have steadily increased during the last decades [1].

Unfortunately, most of these plants are designed on the basis of empirical data and quite often unexpected and unexplainable fluctuations in fermenter performance occur [2], [3] and even recent publications come to the conclusion that the engaged microorganisms still work within a ‘black box’ [4]. However, there has been significant progress in identifying and investigating microbial key players of anaerobic fermentations especially since culture independent techniques have become increasingly available in microbiology [1], [5], [5]–[9].

In a former investigation we could prove *Methanosarcina* sp. to be a key player during thermophilic biogas production – especially during the recovery after disturbed fermentations [7], a finding which corresponds with similar investigations [10], [11]. It was possible to prove that inoculation with *Methanosarcina* sp. could successfully restart or at least accelerate the restoration process after a disturbance of the fermentation [12]. Despite this progress a couple of questions remain unsolved, especially those connected with the microbial succession during optimal and malfunctioning.

Thus, within the present investigation we used different methods to characterize the microbial succession during a batch fermentation. These methods comprised both, fingerprint and analytical approaches and especially focused on the abundance of *Methanosarcina* sp., and its correspondence with the biogas production, as we assumed that a lack of *Methanosarcina* sp. might cause severe disturbance during thermophilic digestion.

## Materials and Methods

### Medium and medium preparation

The synthetic minimal medium described by [7] was used with the modifications that the concentration of NaHCO_3_, carboxymethylcellulose (CMC) and peptone from casein was reduced to 4.2 g (50 mM), 2 g and 2 g per litre, respectively. The third complex carbon source was yeast extract (2 g L^−1^), leading to a total carbon content of 3.18 g L^−1^ medium. The components were weighed in a bottle and dissolved with A. dest. resulting in 5 L medium. After autoclaving the hot medium (>75°C) was immediately transferred with a peristaltic pump into a lab-scale fermenter (see below) while flushing it with N_2_/CO_2_ (7/3) to reduce the contamination with O_2_. Anaerobic conditions were controlled by the redox indicator resazurine and the pH value was set to 7.5 with HCl.

### Inoculum, cultivation conditions and sampling

Sludge of a thermophilic anaerobic plug-flow reactor with an operating volume of 750 000 liters [2] was used as an inoculum. After the transport to the laboratory the sludge was diluted 1∶5 (v/v) with boiled distilled water, which was flushed with pure N_2_ for 10 min during cooling down. The diluted fermenter sludge (DFS) was shaken for 30 min at 100 rpm to allow homogenization. The DFS had a dry matter concentration of approximately 2.5%, which consisted of 65% organic matter. The dried sludge had a total carbon and nitrogen content of about 30% and 2.5%, respectively. For detailed chemical, physical and biological properties of the sludge it is referred to [2]. The 5 L medium was inoculated with 555 mL DFS representing a 1∶10 inoculation.

The fermenter was maintained at 52±0.02°C, moderately stirred with 50 rpm, and sampled 28 times during the whole investigation period of 65 days. At every sampling point 66 mL of the culture broth were withdrawn for subsequent analyses. As no fresh medium was provided due to the batch cultivation mode, a gradual decrease of the liquid volume was apparent with a final total withdrawal of approximately 1.85 L.

### Fermenter system and determination of the gas production

As a fermentation system a software-controlled BIOSTAT Aplus (Sartorius, Germany) fermenter with an operation unit and a working volume of 5 L was chosen. The fermenter had a liquid and gas sampling port, ports for pH adjustment, and a port for gas sparging to get rid of remaining O_2_. The produced biogas had to pass an exhaust cooler to reduce the loss of water vapor, which would be significant under thermophilic conditions. Afterwards the quantitative gas production was evaluated with a Rigamo MilliGascounter (Ritter GmbH, Germany) with a resolution of approximately 3.3 mL. Every time a switch occurred, the software calculated the cumulative gas production and gas production rate. The temperature was maintained by a cooling finger inside the fermenter and a heating blanket around the glass vessel, and the pH was measured online.

### Biogas composition and VFAs

The analysis of the gas quality (H_2_, CO_2_, CH_4_ and O_2_ concentration) was done according to [12]. The samples for the determination of the volatile fatty acids (C_1_–C_7_) were prepared as previously described [12], [13] but the operational settings of the HPLC system LC-20A prominence (Shimadzu) were slightly modified: oven temperature 65°C, flow rate 0.8 mL min^−1^, mobile phase 5 mM H_2_SO_4_ and measurement of absorbance at 210 nm.

### DNA extraction, end-point and quantitative PCR, DGGE and DNA sequencing

Out of selected samples (see below) 700 µL were extracted with a NucleoSpin Soil DNA extraction kit (Macherey-Nagel) and eluated in 50 µL buffer. Quantity and quality of extracted DNA were analyzed in duplicates with a NanoDropTM 2000c spectrophotometer (Thermo Scientific).

For archaea-specific end-point PCR/DGGE (denaturing gradient gel electrophoresis) analysis the primer pair 787F and 1059R (Arc) was applied [14] at which a GC-clamp was attached to the 5′-end of the forward primer [15]. The PCR reaction mix contained 200 µM dNTPs, 0.2 µM of each primer and 0.08% BSA (bovine serum albumin). For end-point amplification a Taq-DNA-Polymerase (BioThermTM) was used and finally 1 µL of template was added. Amplification conditions for archaea detection included an initial denaturation step (5 min, 95°C), 35 cycles of denaturation (45 s, 95°C), annealing (45 s, 57°C) and elongation (45 s, 72°C), and a final elongation step (7 min, 72°C). All PCR products were checked in a 1.5% agarose gel electrophoresis.

For methanogen specific end-point PCR/dHPLC general primers (109f/1492r) [16], [17] and methanogen specific primers (O357f/O691r) [18] were used according to standard protocols. The reaction mixture contained 25 µL MyTaqTM 2×Mix PCR mixture, primers in a final concentration of 0.5 µM, 50 µg bovine serum albumin (aqueous solution, filter sterilized), and PCR grade water to achieve a final volume of 50 µL. Following PCR-programs were used for amplification of DNA: for 109f/1492r an initial denaturation step (10 min, 95°C), 35 cycles of denaturation (30 s, 95°C), annealing (30 s, 52°C) and elongation (45 s, 72°C) and a final elongation step (10 min, 72°C); for O357fGC/691r: an initial denaturation step (10 min, 95°C), 35 cycles of denaturation (30 s, 95°C), annealing (30 s, 49°C) and elongation (30 s, 72°C) and a final elongation step (7 min, 72°C).

All three parallels of the selected sampling points (day 0, 4, 18, 26 and 41) were analyzed in a DGGE and selected reference organisms were analyzed, to compare band patterns with the complex DNA samples to check for possible similarities. The DGGE protocol was altered based on the work of [19]. The acrylamide concentration in the gel was between 7 and 8%, while urea and formamide concentrations were set between 40 to 60%. For the separation an INGENYphorU electrophoresis system was used (60°C, 100 V for 16 h). Afterwards DNA bands were stained with silver nitrate. For the evaluation conserved gels were scanned and analyzed via GelCompare II software (Applied Maths). For quantification of distinct DNA bands densitometric curves of each lane were readout with ImageJ software (available at: http://rsb.info.nih.gov/ij/) after color separation and background subtraction. Afterwards, an averaged threshold was determined; peak areas were defined and set to relation to the sum of peak areas per lane. To gain more detailed qualitative information most representative samples were loaded on a new gel, bands were separated as described above and most abundant bands were isolated. DNA bands were stained with SYBR Gold Nucleic Acid Gel Stain (Invitrogen). Under UV light fluorescing bands were excised, suspended in A. d. and used as template in a further PCR with the archaea-specific primer pair 787F (without GC-clamp) and 1059R. Positive PCR products were purified with NucleoSpin Extract II (Macherey-Nagel) and sequenced by Eurofins MWG Operon. Passed sequences were processed with CLC DNA Workbench 5.6.1 (CLC bio) and aligned via NCBI Blast tool (http://blast.ncbi.nlm.nih.gov/).

For quantifying total archaea, representative for methanogenic archaea, the above mentioned primer pair Arc was applied in a quantitative PCR (qPCR) after evaluation of its specificity and applicability [20]. We used specific primers for both, for archaea and for methanogens but, although both results were very closely correlated, the latter primers were less reliable so that only the data for archaea are presented within this paper.

For amplification the SensiFAST SYBR No-ROX kit (Bioline) was used. The primer concentration was set to 0.2 µM per reaction. Amplification conditions were as following: 35 cycles of repeating denaturation- (20 s, 95°C), annealing- (20 s, 61°C), and elongation- (20 s, 72°C) steps. For quantification of cellulose-degrading microorganisms the primer set cel5 was applied with specifications according to [21]. A Corbett Life Science (Qiagen) Rotor-Gene 6000 system was used for measurements. PCR products were checked with melt curve analysis for specific amplification, absence of primer dimers and melting behavior of products.

### dHPLC and DNA sequencing

dHPLC (denaturing high performance liquid chromatography) was basically carried out as described in [22] using an elution gradient from 50 to 56% buffer B in 24 min. To obtain additional information on the microbial methanogenic community, pure culture amplicons of various methanogenic archaea were used in order to match peaks with the same retention time. In cases of uncertainty but similar retention time, samples were spiked with pure culture amplicons of the nearest peak derived from a pure culture. If a second peak was found, the peak match was rejected. Additionally, peaks of dHPLC separation were collected (using a Shimadzu fraction collection system FRC-10A), liquid volatilized, and an additional PCR was carried out using methanogen specific primers. Subsequently, an aliquot was loaded on to dHPLC to test the presence of only one peak (in order to allow sequencing), else the collection procedure was repeated. When satisfying results were obtained (not possible for all peaks), amplicons were sequenced at MWG Operon (Germany). Sequence comparison and blast search were carried out using CLC Main Workbench 6.7 (CLC bio).

### PLFA analysis

For the analysis of phospholipid fatty acids (PLFA), the samples were extracted following the method described by [23]. The extracts were separated into neutral-, glyco-, and polar lipids by solid-phase extraction on a Strata-Si Column (Phenomenex), and the polar fraction was subsequently transesterified via a modification of the method described by [24]. The PLFAs were analyzed by a GC 2010 (Shimadzu, Japan), equipped with a flame ionization detector (FID) and helium as carrier gas. A fused silica capillary column (Equity-1, 60 m, 0.25 mm inner diameter, 0.25 µm film thickness, Supelco) was used. The injector port was set to 250°C and the FID to 330°C. The employed oven temperature program was: 100°C for 3 min, increase to 300°C at 3°C min^−1^ and hold for 15 min as described in [25].

## Results and Discussion

**Figure 1:**
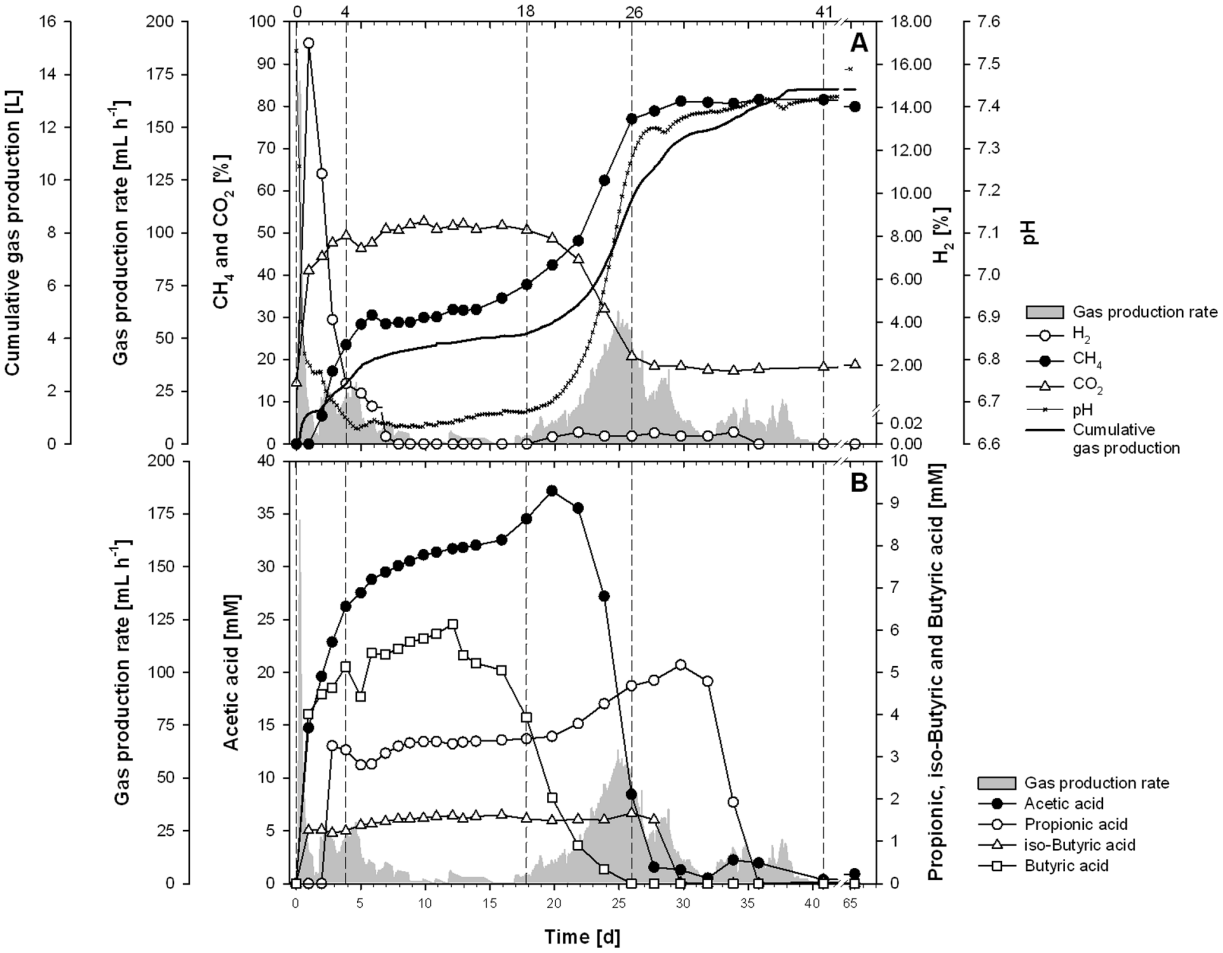
Fermenter performance. pH-values, qualitative and quantitative properties of biogas (A) and concentrations of VFAs (B) during the fermentation. Gas production rate (grey background) is given in A and B to ease the comparison. Dashed lines at t = 0, 4, 18, 26, 41 outline the samples which were additionally investigated by molecular approaches.

Figure 1 shows the course of the batch fermentation with respect to the most important fermenter properties including quality and quantity of biogas. At each of the sampling days 66 mL of the culture broth was withdrawn and kept frozen at −20°C till the whole process was finished. Afterwards, concentrations of VFAs were determined within all samples. On the basis of the process parameters, we decided to investigate samples from t = 0, 4, 18, 26 and 41, with molecular approaches and PLFA analysis in detail. At these days – indicated by dashed lines in Figure 1 – distinct changes in fermentation performance occurred, and thus, differences in microbiology should become obvious.

### Start up

At the very beginning of the fermentation there was a distinct decrease in pH from about 7.5 to 6.6 connected with a sharp increase in the concentrations of H_2_ and CO_2_ in the headspace and the concentrations of acetic and butyric acid in the sludge. Altogether, this obviously reflects the high metabolic activity of hydrolytic and acetogenic microorganisms, resulting in about 40% CO_2_, 17% H_2_, 15 mM acetic acid and 4 mM butyric acid within only one single day. The production of appreciable amounts of iso-butyric acid and propionic acid took more time and concentrations reached approximately 1.5 mM and 3 mM at t = 1 and t = 4, respectively. At these levels the two acids remained remarkably constant till the second phase of high biogas production occurred.

PLFA analyses showed high initial concentrations of long polyunsaturated fatty acids, pointing to a high abundance of eukaryotic cells, possibly deriving from plant material introduced to the fermenter sludge. This explanation seems quite probable as these fatty acids completely disappeared within four days of fermentation.

**Figure 2:**
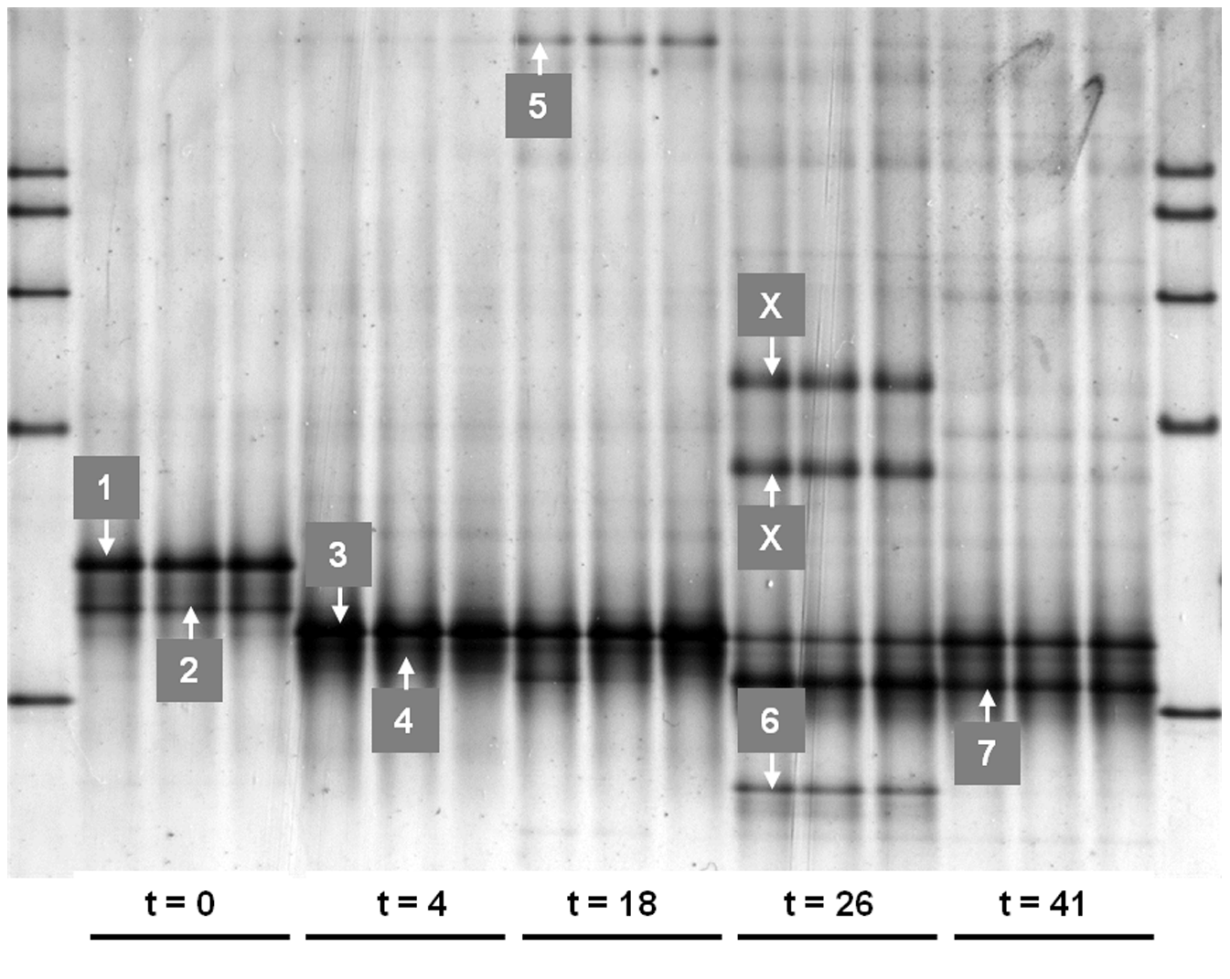
Archaeal DGGE. DGGE of archaeal PCR-products out of samples taken at day = 0, 4, 18, 26 and 41. Assignment of different bands: 1 *Methanothermobacter thermoautotrophicus*, 2 *Methanothermobacter wolfei*, 3 *Methanoculleus thermophilus*, 4 *Methanosarcina* sp., 5 *Thermoplasma* sp., 6 *Methanosarcina thermophila*, 7 *Methanosarcina thermophila*.

Figure 2 shows the DGGE patterns of nucleic acids amplified with archaea-specific primers. Results prove a distinct dominance of *Methanothermobacter thermoautotrophicus* and *Methanothermobacter wolfei* at t = 0. These two members of *Methanothermobacter* as well as all other organisms, which will be discussed within the present paper, exactly matched the reference lines of the respective pure culture (as far as available) and/or were identified by sequencing. The dominance of *Methanothermobacter* sp. confirms previous investigations of the thermophilic fermenter where the inoculum was taken from for the present investigation, and where *M. wolfei* has been proven to be the dominant methanogenic organism [9]. Both species of *Methanothermobacter* are efficient hydrogenotrophic organism (following reaction 1, shown in Table 1) and so this efficient pathway of methanogenesis started after a very short lag phase of not more than two days. This was also proven by a very sharp decrease in the concentration of H_2_, which fell beneath the detection limit (0.005%) again within one week. However, conditions for the initial dominant species seemed to become unfavorable as these organisms completely disappeared till t = 4 and another hydrogenotrophic organism, *Methanoculleus thermophilus*, became increasingly abundant. A very sharp increase in the abundance of *M. thermophilus* was also proven by dHPLC analyses, which again proved to be an efficient fingerprint method for investigating post-PCR mixtures of nucleic acids [22].

**Table 1:** Thermodynamic properties of selected reactions at standard and at *in situ* conditions*).

Reaction	Standard conditions (kJ reaction^−1^)	*In situ* conditions*) (kJ reaction^−1^)
(1) 4H_2_+HCO_3_^−^+H^+^→CH_4_+3H_2_O	−135.5	−43.4
(2) Butyric acid^−^+2H_2_O→2 acetic acid^−^+H^+^+2H_2_	48.2	−8.3
(3) Acetic acid^−^+H_2_O→CH_4_+HCO_3_^−^	−31.0	−20.7
Reaction (1) plus two times reaction (2) resulting in		
(4) Butyric acid^−^+4H_2_O→2CH_4_+2HCO_3_^−^+H^+^+2H_2_	−13.8	−49.7

*) 52°C and real concentrations of gases and VFAs according to [26].

**Figure 3:**
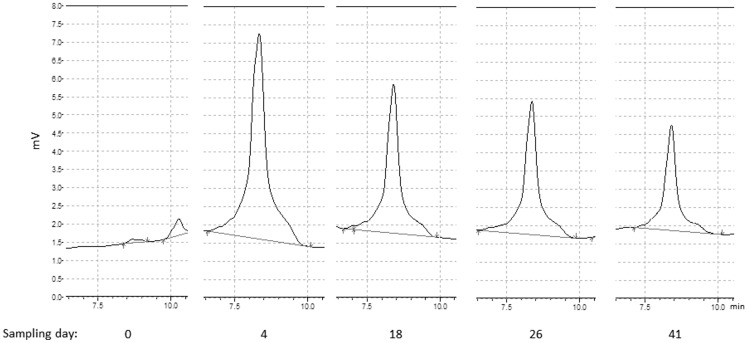
dHPLC of *Methanoculleus* sp. dHPLC signals [mV] of *Methanoculleus* sp. within PCR-products of different samples taken at day = 0, 4, 18, 26 and 41.

Figure 3 shows the course of the abundance of *M. thermophilus* during the whole fermentation. The DNA from the peaks was gathered after HPLC analysis, sequenced and proven to derive from *M. thermophilus* (100% identity). Obviously, this methanogen could rapidly respond to the harsh initial conditions and to a certain extent better handle the steadily increasing concentrations of VFAs accompanied with the decreasing pH. Besides that, the hydrogenotrophic methanogenesis has once again turned out to be more efficient, not only under standard but also under *in situ* conditions (Reaction 1 vs. 3, Table 1) as proven in an earlier investigation [26].

**Figure 4:**
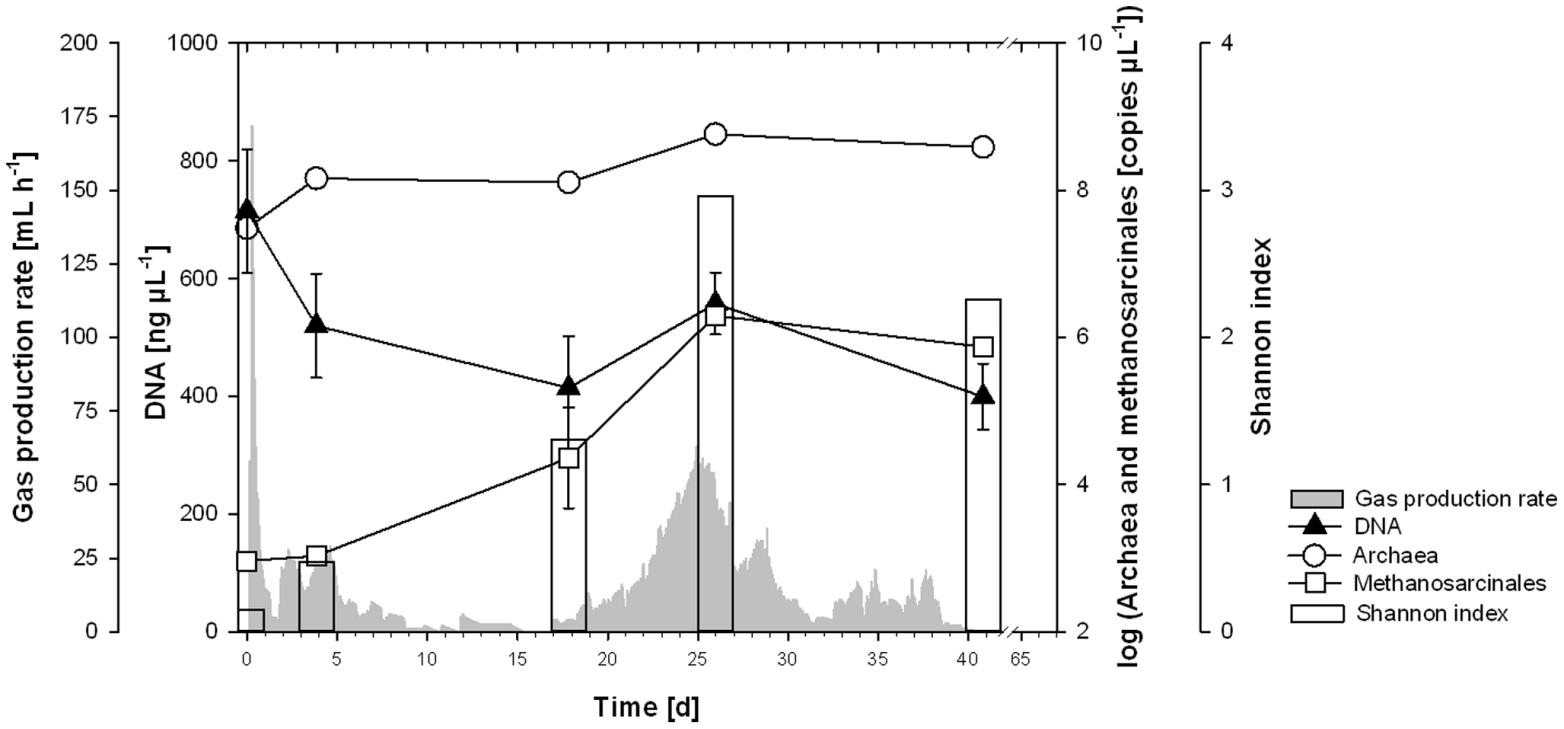
qPCR and archaeal diversity. Content of DNA, copy numbers of Archaea and Methanosarcinales determined via qPCR and Shannon index (bars) on the basis of archaeal DGGE bands before the background of gas production (see Figure 1).

Quantitative analyses proved the total DNA content to be maximal at t = 0, probably because of the above mentioned input of eukaryotic cell material and thus nucleic acids. Contrary, the numbers of total archaea, which we could prove to be equivalent to methanogens in this environment, were minimal at t = 0 and distinctly increased till t = 4 (Figure 4).

### Stagnation

After t = 4 the number of methanogens remained constant till t = 18 (Figure 4) and thus mark a stagnation phase of approximately 14 days, during which nearly no further methane was produced. Concentrations of CH_4_, CO_2_ and H_2_ remained constant at 30%, 50% and <0.005%, respectively, and also the pH remained unchanged at a low level of about 6.7 (Figure 1A). The only parameters which changed within this phase were the concentrations of acetic and butyric acid (Figure 1B). Whereas the first one steadily increased during this whole phase the latter one was characterized by a distinct degradation starting at t = 14. This degradation of butyric acid was nearly the only sign of microbial activity within this phase of fermentation. As the degradation of butyric acid usually follows the reaction 2, shown in Table 1, this degradation should additionally account for the increasing concentration of acetic acid (Figure 1B). It is important to notice that this reaction is endergonic under standard conditions but becomes slightly exergonic under *in situ* conditions (52°C and real pH and concentrations of gases and VFAs) [26]. Furthermore, there is a syntrophic connection with acetate-degrading organisms (e.g. reaction 3, Table 1) so that the sum of the reactions (Reaction 4, Table 1) becomes exergonic, both under standard and even more under realistic conditions. And indeed, the acetic acid oxidation resulted in a distinct production of methane at the end of the stationary phase (Figure 1). When concentration of acetic acids exceeds about 32 mM the oxidation of propionic acid is hampered leading to a second appearance of H_2_ which might favor hydrogenotrophic methanogens.

Microbial analyses proved that *M. wolfei* and *M. thermoautotrophicus* have completely disappeared, that the dominance of *Methanoculleus* sp. steadily decreased and that *Methanosarcina thermophila* very slightly appeared in several strains during this stagnation phase (Figure 2). Obviously, there is a connection between the bad fermenter performance and low gas production during the stagnation phase on the one hand, and the lack of an acetoclastic methanogenic organism, being able to efficiently use the high amounts of acetic acid on the other hand. However, the growth rate of *M. thermophila* was obviously quite low or somehow suppressed, which resulted in a long lag phase of acetoclastic methane production. Although *Methanosarcina* sp. was first detectable at t = 4, it took this strain a long time, till t = 26, until it became dominant. The DGGE band quantification did not only point to the dominance of *M. thermophila* but also to a greater archaeal diversity at t = 26 as several weak and unidentified bands appeared.

Obviously, *M. thermophila* is a very robust acetotrophic methanogen and it was the only one in our investigation which was able to handle high concentrations of acetic acid (35 mM) and the corresponding low pH values, confirming results from the literature [27], [28]. The distinct drop in pH seems to be the reason for the break in CH_4_ production, which only *Methanosarcina* sp. was able to resolve. In former investigations always using the very same inoculum from the 750 000 L large-scale fermenter, sometimes a break with a hampered gas production occurred and sometimes the second phase of gas production was directly connected to the first hydrolytic phase [7], [12]. The reason for this different and hardly predictable behavior is not clear yet but we assume that it corresponds with the presence or lack of *Methanosarcina* sp. Slight differences in buffer capacities of the media resulting in different extents of the pH decreases and thus different growth rates of *Methanosarcina* sp. might be a possible explanation. Another possibility, however not likely, might be that *Methanosarcina* sp. did not grow on acetic acid in the original fermenter sludge and thus had to adapt its metabolism towards the acetoclastic instead of methylo- or hydrogenotrophic pathway leading to a lag-phase. [29] observed that the pregrowth conditions for *Methanosarcina* spp., which define the pathway for methanogenesis, had a significant impact on the occurrence and duration of the lag-phases. Also a very recent investigation proved that bioaugmentation of enriched inocula with *Methanosarcina* sp. led to an improved start-up of digestions suffering from high acetic acid loads [30].

Results from PLFA analyses point to an increase of fatty acids typical for gram positive bacteria and confirm the absence of eukaryotic cells during the stagnation phase (data not shown). These latter results clearly proved that anaerobic fungi like *Neocallimastix* sp., which are sometimes discussed to be engaged in anaerobic digestion [31], [32] should not play any role during the investigated fermentation.

### Second phase of efficient methane production

As mentioned above, at t = 14 the degradation of butyric acid started but it was not before the rapid degradation of acetic acid, started at t = 19, that the second increase in gas production occurred (Figure 1B). Within a few days the concentration of CH_4_ increased to the final concentration of about 80%, whereas the content of CO_2_ decreased to about 15%. Finally the cumulative gas production reached about 14 L standing for about 850 mL gas per gram of carbon, which is a remarkable result compared with gas yields, known from literature [33], [34]. Interestingly, H_2_ became detectable again at the end of the stagnation phase. It should be derived from the degradation of VFAs (see Table 1) and might have promoted the growth of hydrogenotrophic methanogens. As several bands appeared in the DGGE, in all three independent DNA-extractions, and because the number of operons per organisms (probably not more than three) should be constant for a single organism, we think that the bands represent different species of *Methanosarcina sp.* or at least different strains of M. *thermophila*. It is important to keep in mind that *Methanosarcina* sp. is a very versatile methanogen with respect to its substrates because it is able to use all four known methanogenic pathways, which are the hydrogenotrophic, acetoclastic, methylotrophic and the methyl reduction way [11], [35], [36]. Altogether, our results as well as the referred literature, emphasize the potential of *Methanosarcina* sp. as the central key player under high organic loads or deteriorated conditions, as it was the case during this second phase of gas production. Although within complete different habitats – an abandoned coal mine and in a rice field – [37] and [35] could also prove Methanosarcinales to govern CH_4_ formation by utilizing acetic acid rather than H_2_.

Accompanying the distinct degradation of acetic acid, the pH rose again and reached a level of about 7.4. Obviously this was favorable for a greater variety of methanogens, apart from *M. thermophila*. The Shannon index calculated on the basis of DGGE-data proved the highest archaeal diversity at t = 26. Besides *M. thermophila* and *M. thermophilus* a further organism could be identified by all the methods applied, namely *Thermoplasma* sp. or at least some closely-related archaeon representing a non methanogenic organism, which is usually known for its extremophilic way of living [38]. However, despite the occurrence of *Thermoplasma* sp. and despite the high archaeal diversity, *M. thermophila* remained the dominant organism, and also the gas production rate reached its optimum at this time (Figure 1). At t = 26 the concentration of DNA increased again and the number of archaea (determined via qPCR) reached its maximum (Figure 4).

### Final phase

At the end of the fermentation the gas production ceased and the concentrations of VFAs, CH_4_ and CO_2_ were at a constant level. All other parameters describing abundance and activities of the engaged microorganisms distinctly decreased and reached final minima.

The distinct succession from a hydrolytic to a hydrogeno- and acetotrophic community was well documented by DGGE and dHPLC and confirmed by qPCR measurements as well as sequencing data. PLFA analyses in contrast seemed to be of limited evidence in anaerobic systems due to the uncertainties in assignment of specific fatty acids to microbial groups. However, within the present investigation we could prove that there were only very few key players engaged in the investigated digestion, i.e. *Methanothermobacter thermoautotrophicus* and *M. wolfei* at the beginning, *Methanoculleus thermophilus* during the intermediate and to minor quantities in the second phase of high gas production, and *Methanosarcina thermophila* most dominant during the second phase of high gas production.

Members of the genera *Methanosarcina* and *Methanosaeta* are the only methanogens able to degrade acetic acid. While *Methanosarcina* sp. usually dominates at high acetic acid concentrations because of its high conversion rates and low affinity, *Methanosaeta* sp. dominate at opposite conditions due to its high affinity but low conversion rates [10], [11], [30]. In our investigation threshold values for acetoclastic methanogenesis were found to be around 0.6 mM, which corresponds to findings of [39] who determined for *M. barkeri* and *M. mazei* 1.2 and 0.4 mM, respectively, whereas distinct lower values (0.07 mM) were calculated for *Methanosaeta* sp.. Additionally, in an early, anyway excellent work, kinetics of *Methanosarcina* sp. MSTA-1 was investigated [38]. Under optimum temperature and pH conditions Km for acetate kinase and threshold values for acetate were 10.7 and 0.7 mM respectively, thus again confirming our data. Besides, *Methanosarcina* sp. was shown to have a wide pH-range optimum for growth, and slightly acidic conditions even seem to induce increased growth rates [40].

Overall, *Methanosarcina thermophila* seems to be the most important methanogen in the investigated environment, as it was able to terminate a stagnation phase, most probably caused by a decreased pH and accumulated acetic acid. Thus, besides the inoculation with *Methanosarcina* sp., an adaptation of fermenter conditions towards properties favorable for this organism might be a promising possibility to skip phases of low gas production and to optimize CH_4_ yields during anaerobic fermentation. Nevertheless, further research is required, also with respect to potential for up-scaling and applicability.
